# Novel AR/AR-V7 and Mnk1/2 Degrader, VNPP433-3β: Molecular Mechanisms of Action and Efficacy in AR-Overexpressing Castration Resistant Prostate Cancer In Vitro and In Vivo Models

**DOI:** 10.3390/cells11172699

**Published:** 2022-08-30

**Authors:** Elizabeth Thomas, Retheesh S. Thankan, Puranik Purushottamachar, Weiliang Huang, Maureen A. Kane, Yuji Zhang, Nicholas P. Ambulos, David J. Weber, Vincent C. O. Njar

**Affiliations:** 1 Department of Pharmacology, University of Maryland School of Medicine, 685 West Baltimore Street, Baltimore, MD 21201, USA; 2 The Center for Biomolecular Therapeutics, University of Maryland School of Medicine, 685 West Baltimore Street, Baltimore, MD 21201, USA; 3 Flavocure Biotech, 701 E. Pratt St, Ste 2033, Baltimore, MD 21202, USA; 4 Isoprene Pharmaceuticals, Inc., 801 W Baltimore Street, Suite 502J, Baltimore, MD 21201, USA; 5 Department of Pharmaceutical Sciences, University of Maryland School of Pharmacy, Baltimore, MD 21201, USA; 6 Division of Biostatistics and Bioinformatics, University of Maryland, Marlene and Stewart Greenebaum Comprehensive Cancer Center, Baltimore, MD 21201, USA; 7 Department of Epidemiology and Public Health, University of Maryland School of Medicine, Baltimore, MD 21201, USA; 8 Department of Microbiology and Immunology, University of Maryland, Marlene Stewart Greenebaum Comprehensive Cancer Center, Baltimore, MD 21201, USA; 9 Marlene and Stewart Greenebaum Comprehensive Cancer Center, University of Maryland School of Medicine, 685 West Baltimore Street, Baltimore, MD 21201, USA; 10 Department of Biochemistry and Molecular Biology, University of Maryland School of Medicine, Baltimore, MD 21201, USA

**Keywords:** castration-resistant prostate cancer, prostate cancer transcriptome, lead next generation galeterone analog, VNPP433-3β, androgen receptor, AR/AR-V7 degrader, MNK1/2-eIF4E

## Abstract

Prostate cancer (PCa) relies in part on AR-signaling for disease development and progression. Earlier, we developed drug candidate galeterone, which advanced through phase 2-clinical trials in treating castration-resistant PCa (CRPC). Subsequently, we designed, synthesized, and evaluated next-generation galeterone-analogs including VNPP433-3β which is potently efficacious against pre-clinical models of PCa. This study describes the mechanism of action of VNPP433-3β that promotes degradation of full-length AR (fAR) and its splice variant AR-V7 besides depleting MNK1/2 in in vitro and in vivo CRPC models that stably overexpresses fAR. VNPP433-3β directly engages AR within the cell and promotes proteasomal degradation of fAR and its splice variant AR-V7 by enhancing the interaction of AR with E3 ligases MDM2/CHIP but disrupting AR-HSP90 binding. Next, VNPP433-3β decreases phosphorylation of 4EBP1 and abates binding of eIF4E and eIF4G to 5′ cap of mRNA by depleting MNK1/2 with consequent depletion of phosphorylated eIF4E. Finally, RNA-seq demonstrates modulation of multiple pathways that synergistically contribute to PCa inhibition. Therefore, VNPP433-3β exerts its antitumor effect by imposing 1) transcriptional regulation of AR and AR-responsive oncogenes 2) translational regulation by disrupting mRNA-5′cap-dependent translation initiation, 3) reducing AR half-life through enhanced proteasomal degradation in vitro and AR-overexpressing tumor xenografts in vivo.

## 1. Introduction

Notwithstanding the revolutionary advancements in biomedical research and patient healthcare, metastatic castration-resistant prostate cancer (mCRPC) poses a major challenge and remains the second leading cause of cancer-related death in men in the United States and the second most frequently diagnosed cancer among males worldwide [1,2,3]. Dysregulated AR is associated with nearly all known cases of PCa and its expression level has been correlated with poor survival due to the transcriptional upregulation of AR-responsive genes [4]. AR is activated by the binding of androgenic hormones and transcriptionally regulates multiple genes including the ones that regulate the cell cycle. The early stages of the disease are dependent on androgen-mediated AR stimulation but eventually, it becomes androgen-independent as the AR acquires autonomous function and drives disease progression [5,6]. Most patients with metastatic PCa respond to androgen deprivation therapy (ADT) initially, but they inevitably relapse and develop castration-resistant PCa (CRPC) [3,7]. CRPC is the lethal stage of the disease mainly driven by de novo synthesis of androgens within the tumor and other mechanisms wherein AR and its splice variants are key players [8,9]. Additionally, the various roles of estrogen receptors (ER) in PCa are demonstrated and reviewed [10,11].

Previously reported full-length AR signaling inhibitors abiraterone and enzalutamide have significant effects on CRPC and prolong the survival of the patients. Although abiraterone inhibits the enzyme CYP17A1 and blocks residual androgen synthesis and AR signaling [12,13], enzalutamide is a potent antagonist of AR that targets the AR signaling and the consequent tumor progression [14,15]. Despite the initial response, the tumor develops resistance against these otherwise promising drugs by eventually reinstating AR signaling with the intra-tumoral levels of androgens close to that found in the normal prostate gland [8,16,17]. In addition to the upregulation of AR activity by gene amplification, AR mutation, and intra-tumoral androgen synthesis the transcriptional activity of the AR may also become completely ligand-independent in PCa cells [16,18,19]. Thus, the current understanding suggests that the preferred rational approach to the therapeutic intervention of PCa would be reducing the AR levels significantly by targeting its stability and modulating expression and/or activity [20,21,22,23].

The setback suffered in current treatments of CRPC is partly due to the perpetuation of AR signaling even in hormone-refractory disease and therefore AR remains essentially a preferred therapeutic target [24]. However, AR is a negative regulator of eukaryotic translation initiation factor 4E (eIF4E) phosphorylation, and antagonizing AR using AR antagonists will lead to elevated levels of phosphorylation of eIF4E by MAP kinase-interacting kinases 1/2 (MKNK1/2 or Mnk1/2) [25]. In fact, the first-generation AR antagonists such as bicalutamide were inefficient in treating CRPC partly due to the activation of eIF4E by Mnk1/2-mediated phosphorylation [25]. Therefore, a combination of AR and Mnk1/2 inhibitors would be an ideal bet to combat CRPC by obstructing AR-mediated transcription and eIF4E-mediated mRNA 5′ cap-dependent translation. Interestingly, only Mnk1 and Mnk2 are known to phosphorylate eIF4E when both partners are bound to the scaffolding protein eIF4G as part of the translation initiation complex eIF4F [4,26]. Therefore, novel drugs that effectively target AR and Mnk-eIF4E axes concurrently are desirable in combating PCa [4].

We have recently developed galeterone (Gal), a potent drug that concurrently targets AR and Mnk1/2 [27]. Though Gal successfully advanced through phase 2-clinical trials in treating mCRPC, it faced setbacks in the pivotal phase 3 clinical trials due to the limited number of patients studied, and relatively higher clinical doses administered [28]. Consequently, we have synthesized a series of galeterone derivatives, the next generation galeterone analogs (NGGA), and evaluated their potential development to treat all stages of PCa focusing on enhanced efficacy, safety, and minimum effective dose (MED) [29]. Recently, we reported VNPP433-3β, an NGGA with high therapeutic index (TI), lower MED, and compelling therapeutic effects in castration-resistant drug-naive PCa preclinical models in vitro and in vivo [30] and inhibits prostate cancer stem cells [31].

In this study, we report the mechanism of VNPP433-3β, a potent degrader of AR. VNPP433-3β decreases the steady-state availability of AR protein substantially by reducing its half-life through accelerated proteasomal degradation. RNA-seq studies further support the antitumor effect due to the shift in transcriptional activity in favor of PCa inhibition. Furthermore, VNPP433-3β depletes Mnk1/2, thereby inhibiting the phosphorylation of eIF4E. The key advantage of VNPP433-3β is its ability to target multiple signaling and metabolic pathways (AR, eIF4E, Mnk1/2, inhibition of 5′cap-dependent mRNA translation, etc.) that play crucial roles in the development and progression of PCa.

## 2. Materials and Methods

### 2.1. Cell Culture and Reagents

The human PCa cell lines, CWR22Rv1 and LNCaP were procured from ATCC (Manassas, VA, USA) and cultured in phenol red-containing RPMI-1640 media supplemented with 10% heat-inactivated standard fetal bovine serum (FBS, GIBCO) and 1% penicillin-streptomycin (10,000 U/mL, Life Technologies, Carlsbad, CA, USA) at 37 °C and 5% CO_2_. Gal and VNPP433-3β were synthesized in-house as previously described [32] and dissolved in cell culture-grade DMSO. Primary antibodies against human AR (#5153), MNK1 (#2195), eIF4E (#2067), p-eIF4E (#9741), 4EBP1 (#9644), p-4EBP1 (#2855), CHIP (#2080), MDM2 (#86934), HSP90 (#4877), GAPDH (#5174), β-actin (#4970), BAX (#41162), BCL-2 (#15071), Capase9 (#9502), MNK2 (Sigma, M0696), secondary HRP-conjugated anti-rabbit (#7074), and HRP-conjugated anti-mouse (#7076) used in the study were procured from Cell Signaling Technology, USA unless otherwise mentioned. CHX, DHT, MG132 and all fine chemicals were purchased from Sigma Aldrich, USA. A full-length AR-overexpressing plasmid vector was purchased from OriGene Technologies, Inc., Rockville, MD, USA.

### 2.2. Molecular Docking

The crystalline structure of the human AR ligand binding domain (LBD) available in the protein databank PDB (PDB id 1E3G, [33]) was employed for docking the ligand VNPP433-3β to the receptor using the algorithm Autodock VINA 1.1.2 [34]. The co-crystallized ligand and water molecules were removed and polar Hydrogens and Kollman charges were added to the protein prior to docking. VNPP433-3β was docked to the ligand binding site of the AR LBD, centering the grid box at 2.135, 27.699, and 3.831 for x, y, and z, respectively, with grid points of 40 each in x, y and z dimensions and default spacing. Rigid docking was performed employing a genetic algorithm with exhaustiveness of conformational sampling set at eight and other parameters at default. Binding pose with the least Gibb’s free energy of binding (ΔG°) and thus the highest binding affinity was selected, and simulations were processed in BIOVIA Discovery Studio Visualizer v21.1.0.20298, Dassault Systemes, San Diego, CA, USA.

### 2.3. Fluorescence Spectroscopy

Fluorescence spectral measurements were carried out using recombinant human AR LBD (Abcam, Cambridge, UK) in the Tecan Spark microplate reader (Tecan Group Ltd., Männedorf, Switzerland) as described previously [35]. Briefly, 10 μM VNPP433-3β was pre-incubated with increasing concentration of AR LBD (0.5, 1, 2.5, 5, 10, 25, 50, 100, 250 nM) at 25 °C for 30 min. The protein-ligand complex was excited at 270 nm and emitted fluorescence spectrum was recorded at λ 300–500 nm. Fluorescence due to tryptophan in the protein was measured in all concentrations of AR LBD in the absence of ligand and the same was subtracted for obtaining the final reading. Change in fluorescence upon protein-ligand interaction, ΔF was plotted as a function of the concentration of AR using GraphPad Prism version 5.0 (GraphPad Software, San Diego, CA, USA). The data was analyzed, and K_D_ values were derived using ‘one-site specific binding’ function in non-linear regression of GraphPad Prism. All experiments were done in triplicates independently.

### 2.4. Cellular Thermal Shift Assay (CETSA)

Thermal stabilization of protein upon ligand binding was quantitatively measured using Cellular thermal shift assay and detected using western blotting as described previously [36]. Briefly, LNCaP cells (~3 million cells/mL) were exposed to 10 µM VNPP433-3β for 4 h, harvested, and resuspended in 1 mL of PBS supplemented with protease inhibitors. The cell suspension was distributed 100 µL (~3 million cells) each into 0.2 mL PCR tubes and heated at a designated temperature for 3 min in a thermal cycler (Applied Biosystems, Waltham, MA, USA). Immediately after heating, the tubes were incubated at room temperature for 3 min followed by snap-freezing in liquid nitrogen. Cells were lysed by tandem freeze-thaw cycles thrice in liquid nitrogen or heating block set at 25 °C and tubes are vortexed briefly after each thawing step. Subsequently, the cell lysates were centrifuged at 20,000× *g* for 20 min at 4 °C to pellet down the cell debris, precipitated, and aggregated proteins. Supernatant fraction containing the soluble protein was subjected to immunoblotting and the relative intensity of the bands was quantified using Image lab (Bio-Rad, Hercules, CA, USA) and plotted as a function of temperature to yield the apparent melting curve.

### 2.5. Immunoblotting

Cells were lysed with radioimmunoprecipitation assay (RIPA) buffer supplemented with 1X protease inhibitors (Roche, Indianapolis, IN, USA), phosphatase inhibitors (Thermo Scientific, Waltham, MA, USA), 1 mmol/L EDTA and 1 mmol/L PMSF (Sigma) and immunoblotting analyses were performed as described previously [37,38]. For cycloheximide (CHX) chase studies, hormone-starved CWR22Rv1 and LNCaP cells were treated with CHX (10 μM) in presence of VNPP433-3β (10 µM), Gal (10 µM), or vehicle. Cells were collected at indicated time points for immunoblot analysis and relative AR protein expression was quantified and plotted. Androgen-deprived cells were treated with DHT (1 or 10 nM) in the presence or absence of VNPP433-3β (10 µM) and examined AR protein level by immunoblot analysis. All experiments were performed in triplicates.

### 2.6. Co-Immunoprecipitation (Co-IP) Assay

CWR22Rv1 and LNCaP cells were hormone-starved for 48 h, treated with VNPP433-3β (10 µM) for 4 h. Immunoprecipitation was performed using Dyna beads conjugated to Protein A in accordance with the manufacturer’s instructions (Thermo Fisher Scientific, Waltham, MA, USA). Briefly, 50 μL Dyna beads conjugate was incubated with 3–5 μg of antibody for 10 min. Dyna beads-Ab complex was then washed in washing buffer and subsequently binding buffer. Cell lysate containing 1mg protein was added to the Dyna beads-Ab complex and incubated for 10 min. The complex was washed thrice using washing buffer and resuspended in elution buffer followed by immunoblotting with the appropriate antibody. To study protein-protein interactions, hormone-starved CWR22Rv1 and LNCaP cells were treated with VNPP433-3β (10 µM) for 4 h and the cell lysates were incubated with Dyna beads-Ab complex following the manufacturer’s instructions and immunoblotted.

### 2.7. mRNA 5′ Cap (m^7^GTP) Binding Assay

CWR22Rv1 and LNCaP cells seeded in 6-well plates 24 h prior to drug exposure were treated with VNPP433-3β (10 and 20 μM) for 24 h. Cells were then washed with ice-cold PBS and total cell lysates were prepared in cap-binding buffer containing 150 mM NaCI, 50 mM Tris, pH 7.5, 50 mM NaF, 10 mM Na-pyrophosphate, 1 mM EDTA, and 2.5 mM Na-orthovanadate supplemented with protease inhibitor cocktail (Sigma) at 4 °C for 15 min. Subsequently, 25 μL of γ-Aminophenyl-m^7^GTP (C10-spacer)-Agarose beads (Jena Bioscience, Jena, Germany) was added to 500 μg of total proteins and incubated at 4 °C for 2 h with constant shaking. Samples were then washed with cap-binding buffer, quenched with sample buffer, and subjected to immunoblotting.

### 2.8. RNA-Sequencing and Transcriptome Analyses

CWR22Rv1 cells were treated with 10 μM VNPP433-3β for 24 or 48 h in triplicates. Cells were then washed with PBS and RNA was extracted using RNAeasy Plus mini kit (Qiagen, Hilden, Germany). The concentration and quality of total RNA samples were assessed using Agilent 2100 Bioanalyzer, and a RIN (RNA Integrity Number) threshold of 8 or above was employed for all samples. RNA-Seq libraries were prepared with the NEB Ultra II Directional RNA library prep kit. The libraries were assessed for quantity and size distribution using Qubit and Agilent 2100 Bioanalyzer. Sequencing was performed on an Illumina NovaSeq S2 PE100 bp lane at Maryland Genomics, Institute for Genome Sciences, University of Maryland Baltimore. Phred quality score (Q score) was used to measure the quality of sequencing. More than 90% of the sequencing reads reached Q30 (99.9% base call accuracy). QIAGEN Ingenuity Pathway and Gene Set Enrichment analyses (GSEA) were carried out to reveal canonical cellular pathways modulated by VNPP433-3β.

### 2.9. siRNA-Mediated Knockdown of Gene Expression

Specific siRNA targeting the gene of interest and scramble siRNA (siControl) were purchased from Ambion (Foster City, CA, USA). CWR22Rv1 and LNCaP cells were grown in 6-well culture plates and transfected with specific siRNA using lipofectamine RNAiMax transfection reagent (Invitrogen, Waltham, MA, USA) in Opti-MEM reduced serum medium (Thermo Fisher Scientific, USA) for 48 h following the manufacturer’s instructions. Scrambled siRNA was transfected as control and knockdown was confirmed by immunoblot analyses.

### 2.10. In Vivo Tumor Xenograft Studies

The castration-resistant prostate cancer (CRPC) cell line 22Rv1 that stably overexpresses full-length AR and inherently expresses fAR and AR-V7 was used to induce tumor xenografts in immunodeficient male NRG mice. The mice (age 5–7 weeks) were procured from the Veterinary Resources, University of Maryland School of Medicine (Baltimore, MD, USA), housed under sterile conditions, and fed with sterile pellets and water ad libitum. After a week of acclimatization, 5 × 10^6^ 22Rv1 cells that stably overexpress full-length AR were subcutaneously injected into the left flank of mice. After three weeks of inoculation and upon reaching the tumor volume of ~100 mm^3^, the mice were randomized into five groups of five animals. Mice-bearing tumors of 22Rv1 cells that are transfected with an empty plasmid vector served as transfection control and mice bearing AR-overexpressing 22Rv1 tumor xenografts served as treatment control. Both the control groups of mice were orally administered with vehicle (2% β-cyclodextrin in saline, PO). The other three groups of mice bearing AR-overexpressing 22Rv1 tumor xenografts were orally administered with galeterone (100 mg/kg; twice a day) or VNPP433-3β (10 or 20 mg/kg; once a day), five days a week for 17 days. The animals were monitored daily for general health and body weight was recorded three times a week. The tumor size was measured thrice a week using digital calipers and tumor volume was calculated using the formula length × width 2 × 0.5 (mm^3^). Upon reaching the tumor length close to 20 mm in control groups (17th day), the experiment was terminated, the animals were euthanized, and tumors were excised for further immunoblot and histology analyses as described previously [39]. All in vivo studies in mice were performed in accordance with the humane use of experimental animals following review and approval by the Institutional Animal Care and Use Committee (IACUC), University of Maryland School of Medicine, Baltimore, MD, USA, per IACUC No. #0,421,007 dated 31 January 2022.

### 2.11. Statistical Analysis

Statistical comparisons were made by one-way ANOVA followed by Student’s *t* test using GraphPad Prism 5.0 software (GraphPad Software, Inc., La Jolla, CA, USA). A probability value with * *p* < 0.05, ** *p* < 0.01 and *** *p* < 0.001 were considered statistically significant. As specified in the figure, values in data are expressed as the mean  ±  SEM of three or more independent experiments.

## 3. Results

### 3.1. RNA-Seq Demonstrates Modulation of Multiple Pathways Leading to PCa Inhibition by VNPP433-3β

**Figure 1:**
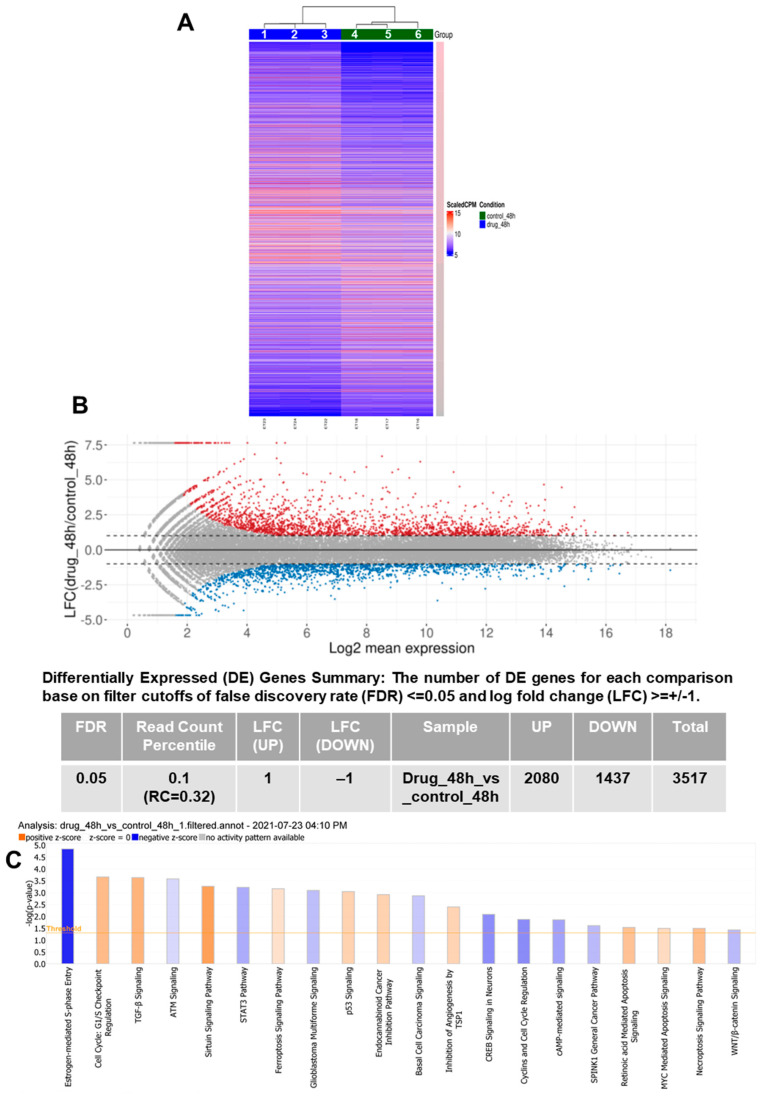
**RNA-seq and transcriptome analyses demonstrate modulation of several pathways in favor of PCa inhibition upon treating CWR22Rv1 cells with VNPP433-3β**. (**A**) Heatmap of the differentially expressed genes in 10 µM VNPP433-3β-treated CWR22Rv1 cells (lanes 4–6) for 48 h compared to the control (lanes 1–3), illustrating the clustering for each comparison of interest based on the Gene Expression Counts and Log Fold Change (LFC) for the Differentially Expressed Genes (DEGs). Columns correspond to samples and rows correspond to genes. Scale bar represents a graded change in LFC where pink is higher LFC, gray is lower LFC. Gene expression values were derived from DESeq/DESeq2 normalized counts. (**B**) MA Plot showing the relationship between expression change (LFC, M) and average expression (log2 mean expression, A). Red points indicate the upregulated genes with FDR ≤ 0.05 and LFC ≥ 1 and blue points indicate downregulated genes with false discovery rate (FDR) ≤ 0.05 and log2-fold-change ≥ ±1. Dashed lines indicate the LFC cutoff values of ±1. Points colored in gray indicate the genes that are not significantly differentially expressed. Table shows the number of genes differentially expressed. (**C**) QIAGEN Ingenuity Pathway Analysis shows that VNPP433-3β steered the transcription of multiple canonical pathways genes in favor of PCa inhibition. A positive z-score (orange bars) indicates the activation of the given pathway, and a negative z-score (blue bars) reflects the suppression of that pathway.

RNA sequencing and transcriptome analyses in CWR22Rv1 cells reveal that VNPP433-3β triggers significant changes in the cellular transcriptome (Figure 1A). The 10 µM treatment for 48 h resulted in differential expression of 3517 genes, 2080 were upregulated and 1437 downregulated (Figure 1B). Excitingly, the transcription of AR itself is decreased two-fold upon treatment with VNPP433-3β besides the downregulation of several genes involved in oncogenesis including eIF4E (Table S1). Further, to complement this effect, various genes involved in autophagy, apoptosis, ferroptosis, and cell cycle checkpoint were upregulated. We next used the RNA-seq data to evaluate several canonical metabolic and signaling pathways using QIAGEN Ingenuity Pathway Analysis software. More than 20 pathways associated with PCa were significantly modulated by VNPP433-3β (Figure 1C). Notably, the autophagy and cell cycle checkpoints such as p53 signaling were augmented besides activating the regulation of EMT and Sirtuin pathway to maintain genomic integrity. Entry into the S-phase of the cell cycle is significantly inhibited by VNPP433-3β in addition to impeding several oncogenic pathways such as WNT signaling, cyclin and cell cycle regulation, c-AMP mediated signaling, STAT3 pathway, glioblastoma multiforme and basal cell carcinoma signaling, CREB signaling, etc. Therefore RNA-seq data further validates the PCa-inhibiting effects of VNPP433-3β observed in the biochemical studies besides revealing the significant pathways that are modulated. RNA-seq demonstrates a significant shift in transcriptional activity of PCa cells in favor of PCa inhibition.

### 3.2. VNPP433-3β-Induced Degradation of fAR and AR-V7 Is Attributed to the Physical Interaction of VNPP433-3β with fAR

**Figure 2:**
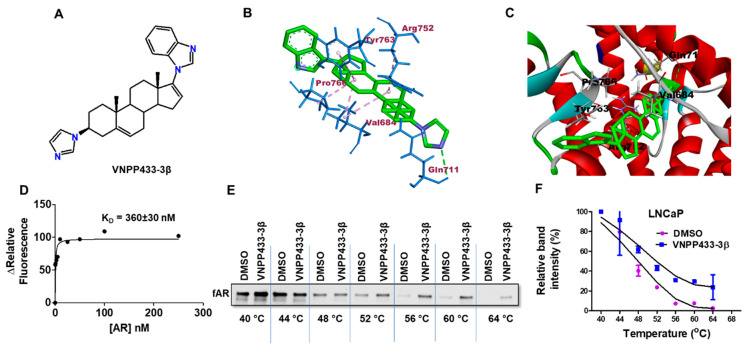
**Molecular interaction of VNPP433-3β with AR.** (**A**) Chemical structure of VNPP433-3β. (**B**) Molecular docked pose of VNPP433-3β in the ligand binding domain of AR showing the interacting amino acid residues. Gibb’s free energy of binding (ΔG°) = −8.3 kcal/mol. (**C**) Protein surface and ligand-binding groove of the AR bound with VNPP433-3β. (**D**) Change in relative fluorescence (ΔF) of VNPP433-3β upon binding AR LBD as a function of increasing concentration of AR LBD. (**E**) Thermal stabilization of AR protein upon ligand binding was measured by CETSA in LNCaP cells. Representative western blot of CETSA-AR in LNCaP cells treated with VNPP433-3β (10 µM). (**F**) Apparent melting curve of AR: graph was plotted as a function of temperature used in heat treatment. All experiments were performed three times independently and data is given as average ± S.E.M. The solid lines represent the best fits of the data to the Boltzmann sigmoid within the GraphPad Prism software.

As the primary studies indicated that AR is the prime target of VNPP433-3β (Figure 2A), we further investigated the likelihood of physical interaction between AR and VNPP433-3β. To shed more light on the interaction of VNPP433-3β with the fAR, we simulated the molecular interaction using the algorithm Autodock VINA [34]. Interestingly, the best model of AR ligand binding domain (LBD)-VNPP433-3β interaction predicted by the algorithm possesses Gibb’s free energy of binding (ΔG°) –8.3 kcal/mol. The substantial negative change in ΔG° suggests that a spontaneous AR-VNPP433-3β interaction is highly favorable thermodynamically. The ligand was docked to the binding pocket identified in the previous crystallographic studies [33] and primarily through the amino acids at the active sites Tyr763, Arg752, Pro766, Val684, and Gln711. Although Gln711 formed H-bonding with the ring N in the ligand, the other four amino acids formed alkyl or pi-alkyl interactions (Figure 2B). The ligand binding was further enhanced by the van der Walls force of attraction contributed by other nine amino acids Ala748, Pro682, Lys808, Met745, Val715, His714, Val685, Gly683, and Asn756. Ligand-binding groove on the protein surface with VNPP433-3β bound to the AR LBD is shown in Figure 2C. Five of the amino acids in the binding site identified in this study are the same ones identified in the crystallographic studies [33]. Further, the VNPP433-3β-AR interaction was studied using purified AR LBD and determined its dissociation constant (K_D_) in vitro by fluorescence spectroscopy. Changes in relative fluorescence upon AR LBD-VNPP433-3β interaction, ΔF was plotted as a function of the concentration of AR (Figure 2D). VNPP433-3β displayed a strong binding affinity for the purified AR LBD with a dissociation constant (K_D_) of 360 ± 30 nM.

We next validated the in-silico and in vitro findings of AR-VNPP433-3β interaction by performing a cellular thermal shift assay (CETSA) in LNCaP cells. The protein level of AR was monitored by western blot (Figure 2E) and relative intensities were plotted as a function of temperature to generate the apparent melting curve (Figure 2F). The results show the presence of intact protein at lower temperatures in untreated cells, but it disappears gradually as the temperature rises to 64 °C. Nevertheless, the VNPP433-3β-treated cells showed a significant level of intact protein that was thermally stabilized by the ligand at 64 °C. This clearly suggests the stabilization of AR by binding to VNPP433-3β thereby preventing its denaturation and ensuing precipitation even at elevated temperatures as high as 64 °C. The results in LNCaP cells emphatically suggest that VNPP433-3β indeed binds fAR and stabilizes it from denaturation. Therefore, different lines of experimental evidence suggest that VNPP433-3β can indeed engage AR within the cells. However, AR-V7 lacks the LBD, and we are investigating a possible alternative mechanism of degradation that may overlap with the model presented for fAR. A recent study demonstrates that AR-V7 functions differently and is independent of fAR [40].

### 3.3. VNPP433-3β Decreases the Half-Life of fAR and AR-V7 by Enhancing the Rate of Degradation

**Figure 3:**
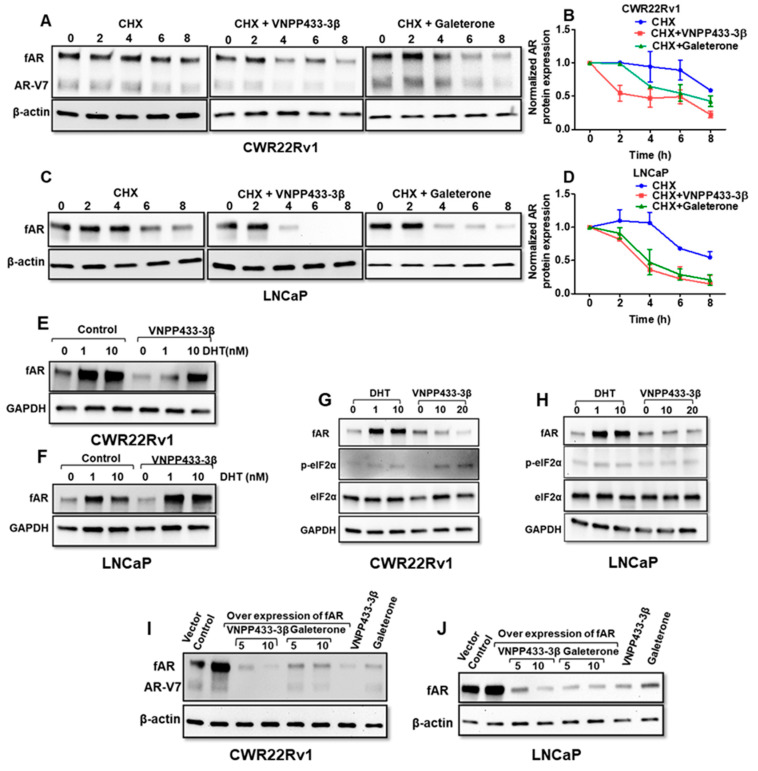
**Evaluation of half-life of AR in presence of VNPP433-3β using eukaryotic translation inhibitor cycloheximide (CHX).** Androgen-deprived CWR22Rv1 (**A**) and LNCaP (**B**) cells were treated with 10 μM CHX and 10 μM VNPP433-3β or vehicle control for the duration as indicated (0, 2, 4, 6, 8 h), 40 μg protein was separated by SDS-PAGE and subjected to immunoblotting. (**C**,**D**) AR protein expression was quantified and normalized with β-actin and plotted. Androgen-deprived CWR22Rv1 (**E**) and LNCaP (**F**) cells were treated with DHT (1 or 10 nM) in presence or absence of VNPP433-3β (10 μM) overnight and immunoblotted for AR and GAPDH. Androgen-deprived CWR22Rv1 (**G**) and LNCaP (**H**) cells were treated with DHT (1 or 10 nM) in the presence or absence of VNPP433-3β (10 μM) overnight and lysates were immunoblotted for AR, p-eIF2α, eIF2α, and GAPDH. CWR22Rv1 (**I**) and LNCaP (**J**) cells were transfected with a plasmid that overexpresses full-length AR and treated with VNPP433-3β or Gal (5, 10 μM) for 24 h and immunoblotted for AR.

Physiologically, the non-ligand bound AR is degraded by a proteasome-dependent pathway, but its stability is increased by androgen binding [41]. This prompted us to investigate the half-life of fAR and AR-V7 in presence of VNPP433-3β in these cell lines. The cells were treated with VNPP433-3β or Gal in presence of cycloheximide (CHX) that blocks new protein synthesis. As shown in Figure 3A,C, treatment with VNPP433-3β decreased the half-life of both fAR and AR-V7 markedly in CWR22Rv1 and fAR in LNCaP cells, respectively. The rate of AR degradation in CWR22Rv1 cells was significantly enhanced by three times following treatment with VNPP433-3β compared to the vehicle control. Although the untreated cells exhibited a half-life of 8.5 h, the treated cells registered a half-life of only 2.7 h in CWR22Rv1 cells (Figure 3B). Similarly, VNPP433-3β decreased the half-life of endogenous AR in androgen-deprived LNCaP cells from 8.5 to 3 h whereas Gal treatment decreased it to 5.8 and 4 h in CWR22Rv1 and LNCaP cells, respectively (Figure 3D). This clearly implies that VNPP433-3β increased the rate of AR degradation at least three times in both the cell lines tested and reduced its half-life significantly.

Further, to ascertain whether the effects of VNPP433-3β were direct and due to AR binding the ligand, we tested if dihydrotestosterone (DHT), the natural ligand of AR could prevent the decline in AR protein. Interestingly, we observed that DHT could partly impede the VNPP433-3β-mediated decrease in AR level in CWR22Rv1 (Figure 3E) and LNCaP (Figure 3F) cells in a dose-dependent manner. This also suggests that VNPP433-3β binds to the AR in the same binding pocket as the natural ligands and the observation is consistent with the docking studies’ results. Since Gal has been reported to activate ER stress response at very high concentrations [42,43], we next studied if VNPP433-3β induces any ER stress response in vitro and affects AR expression indirectly. An increase in phosphorylated eukaryotic initiation factor 2α (p-eIF2 α) is considered a marker of ER stress [44]. Interestingly, we found that level of p-eIF2α did not change in the LNCaP cells in response to VNPP433-3β treatment even at 20 µM (Figure 3H). In slight contrast, though there was no significant change in the CWR22Rv1 cells at 10 µM, a feeble increase is observed in 20 µM of VNPP433-3β (Figure 3G). These results together suggest that VNPP433-3β directly enhances AR protein degradation in LNCaP and CWR22Rv1 cells with no significant stimulation of ER stress.

Next, we overexpressed fAR in CWR22Rv1 and LNCaP cell lines and assessed the effect of VNPP433-3β and Gal in AR-overexpressing cell lines. We found that overexpression of AR did not significantly alleviate the effect of VNPP433-3β or Gal as the levels of fAR and AR-V7 were decreased significantly (Figure 3I,J).

### 3.4. VNPP433-3β Promotes fAR/AR-V7 Degradation by Enhancing Its Interaction with MDM2/CHIP and Disrupting fAR/AR-V7-HSP90 Interaction

**Figure 4:**
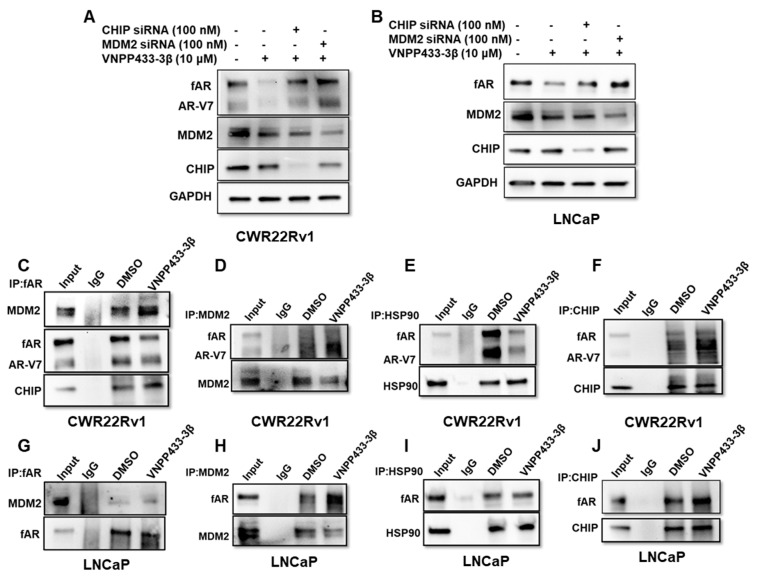
**VNPP433-3β triggers AR/AR-V7 degradation by promoting enhanced interaction of fAR with E3 ligases MDM2 and CHIP.** (**A**) CWR22Rv1 and (**B**) LNCaP cells were transfected with siRNA of MDM2 or CHIP (100 nM, Ambion) for 48 h and exposed to VNPP433-3β (10 µM) for 24 h and the lysates were immunoblotted for AR, MDM2, CHIP, and GAPDH. Scrambled siRNA served as control. CWR22Rv1 (**C**–**F**) and LNCaP (**G**–**J**) cells were hormone-starved for 48 h and treated with DMSO or VNPP433-3β for 4 h. Cell lysate containing 1 mg protein was subjected to immunoprecipitation and subsequent immunoblotting for AR, MDM2, HSP90, and CHIP.

Considering ubiquitin-dependent proteasomal degradation is the major and well-known pathway of AR degradation, we then investigated the role of major proteins operating in the pathway and how VNPP433-3β modulates their activity. Firstly, MDM2 or CHIP was knocked down in CWR22Rv1 and LNCaP cell lines using siRNA prior to treating with 10 µM VNPP433-3β for 24 h. Strikingly, the degradation of fAR and AR-V7 by VNPP433-3β was impaired when either of these E3 ligases was downregulated in CWR22Rv1 (Figure 4A) and LNCaP cells (Figure 4B). The effect was more prominent in MDM2-downregulated CWR22Rv1 cells wherein both fAR and AR-V7 are expressed (Figure 4A). It is worth noting that CWR22Rv1 is an androgen-independent, fast-dividing, and highly tumorigenic cell line. The decline in VNPP433-3β mediated degradation of fAR and AR-V7 was prominent in CHIP-downregulated cells as well, albeit slightly lower than that of the MDM2-downregulated cells.

Intrigued by these results, we then investigated if these proteins physically interacted with AR and if so, this interaction was modulated by VNPP433-3β. To study this, a series of co-immunoprecipitation (co-IP) experiments were carried out. IP of fAR followed by western blot shows that MDM2 and CHIP were co-precipitated with AR, wherein VNPP433-3β treated cells resulted in enhanced interaction of AR with MDM2 and CHIP compared to the vehicle control in CWR22Rv1 (Figure 4C) and LNCaP cells (Figure 4G). Similarly, the VNPP433-3β induced enhanced interactions were further validated by IP using MDM2 followed by western blot for AR in CWR22Rv1 (Figure 4D) and LNCaP cells (Figure 4H). Likewise, the IP of CHIP revealed an enhanced interaction between CHIP and fAR and AR-V7 in CWR22Rv1 cells (Figure 4F) and fAR in LNCaP cells (Figure 4J). The findings suggest that VNPP433-3β indeed enhances the interaction of fAR and notably AR-V7 with the E3 ligases MDM2 and CHIP, thereby strengthening the hypothesis of VNPP433-3β-mediated fAR and AR-V7 degradation.

Heat shock protein 90 (HSP90) is a molecular chaperone that aids other proteins to fold into native conformation. It stabilizes the client proteins against various stresses in the cell and is a key regulator of proteostasis. The HSP90 is found to interact with AR, thereby enhancing the stability of non-ligand bound receptors. Inhibitors of HSP90 such as 17-AAG prevent HSP90-AR interaction, subsequently leading to AR degradation [45]. This prompted us to investigate if VNPP433-3β influences the interaction between AR and HSP90. Intriguingly, immunoprecipitation of HSP90 studies showed that VNPP433-3β indeed disrupted the HSP90-AR interaction, rendering AR available for degradation in CWR22Rv1 (Figure 4E) and LNCaP cells (Figure 4I). The findings of the immunoprecipitation studies further corroborated our siRNA experimental outcomes that suggested CHIP and MDM2 E3 ligases play a crucial role in VNPP433-3β induced fAR/AR-V7 ubiquitination and subsequent degradation.

The co-immunoprecipitation (Co-IP) with AR confirmed the VNPP433-3β-mediated augmentation of interaction between fAR/AR-V7 and E3 ligases MDM2 and CHIP while disrupting the interaction between fAR/AR-V7 and HSP90, thereby compelling fAR/AR-V7 to enter the ubiquitin-dependent proteolysis pathway. This model of AR degradation is consistent with the previous reports of small molecule-induced AR degradation through ubiquitin-dependent proteasomal degradation [24,45]. As this study addresses some of the key components of the ubiquitin-dependent proteasomal degradation, the results firmly suggest that VNPP433-3β indeed triggers enhanced degradation of fAR and its splice variant AR-V7.

### 3.5. VNPP433-3β Abates Binding of eIF4E and eIF4G to 5′ Cap of mRNA Thereby Imposing Translational Regulation

**Figure 5:**
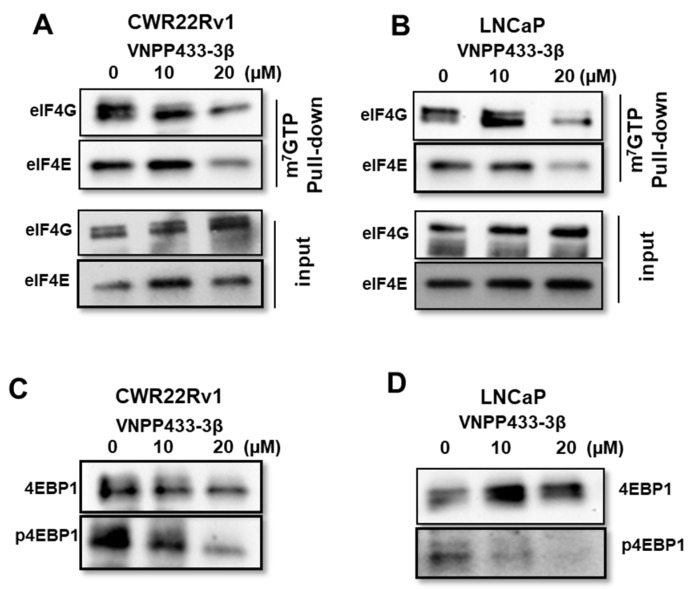
**VNPP433-3β enhances ubiquitin-dependent proteasomal degradation of fAR and AR-V7.** (**A**,**B**) VNPP433-3β abates binding of eIF4E and eIF4G to 5′ cap of mRNA and imposes translational regulation. The mRNA 5′ cap binding assay was performed using m^7^GTP-Agarose beads in total cell lysates of VNPP433-3β-treated (**A**) CWR22Rv1 and (**B**) LNCaP cells and pulled down by centrifugation and subsequently immunoblotted for eIF4E and eIF4G. (**C**) CWR22Rv1 and (**D**) LNCaP cells were treated with vehicle (control) and indicated concentrations of VNPP433-3β (10 and 20 μM) for 24 h and lysates were immunoblotted for 4EBP1 and p-4EBP1.

A key checkpoint in eukaryotic gene expression is translational regulation wherein the formation of the translation initiation complex (eIF4F), the very first step of translation is tightly regulated. Eukaryotic translation initiation factor 4E (eIF4E) is a vital effector molecule and its availability is a rate-limiting factor in the initiation of protein synthesis [4,46]. It binds to the 7-methylguanosine 5′ cap of the mRNA along with eIF4G and eIF4A to form the translation initiation complex eIF4F, thereby recruiting small ribosomal subunit 40S to the mRNA. As VNPP433-3β impaired phosphorylation of eIF4E, we tested if it affects mRNA-5′cap-dependent translational machinery. CWR22Rv1 and LNCaP cells were treated with 0, 10, and 20 µM VNPP433-3β for 24 h, and total cell lysate was subjected to m^7^GTP-Agarose pull-down followed by western blot analyses of eIF4E and eIF4G. Interestingly, the treatment with VNPP433-3β significantly disrupted the mRNA-5′cap-binding ability of eIF4E and eIF4G in a dose-dependent manner in both the cell lines tested (Figure 5A,B). This is a remarkable finding as it interrupts deregulated translational activity of PCa cells. Moreover, studies have shown that the protein level of AR is also regulated at the translational level by modulating the mRNA-5′cap-dependent translation process [47].

Physiologically, eIF4E is sequestered from binding the mRNA by another key protein 4EBP1. However, when 4EBP1 is phosphorylated (p-4EBP1) at one or more of the seven residues known to be phosphorylated by mTOR or yet-to-be-identified other kinases, it gradually loses affinity for eIF4E and liberates eIF4E, which can bind the mRNA 5′-cap, thereby initiating translation [46]. The hyperphosphorylated 4EBP1 has the least affinity for eIF4E and thus it emancipates eIF4E, thereby favoring active translation within the cell. Hence, we tested the total level of 4EBP1 and p-4EBP1 by western blot using a p-4EBP1-specific antibody upon treating the cells with VNPP433-3β. Strikingly, we observed a dose-dependent decrease in p-4EBP1 in response to the increasing concentrations of VNPP433-3β in CWR22Rv1 and LNCaP cells (Figure 5C,D). However, the total protein level of 4EBP1 did not change significantly upon treatment. This observation is consistent with the previous reports that 4EBP1 activity in the cell is mainly regulated at the post-translational level by phosphorylation-dephosphorylation cycles [46]. The finding is encouraging and consistent with the results obtained in the mRNA cap-binding assay. Together these results strongly imply that VNPP433-3β indeed impedes the active protein synthesis, a pre-requisite for the actively proliferating PCa cells.

### 3.6. VNPP433-3β Targets AR In Vivo with Concomitant Tumor Inhibition in CRPC Tumor Xenograft

**Figure 6:**
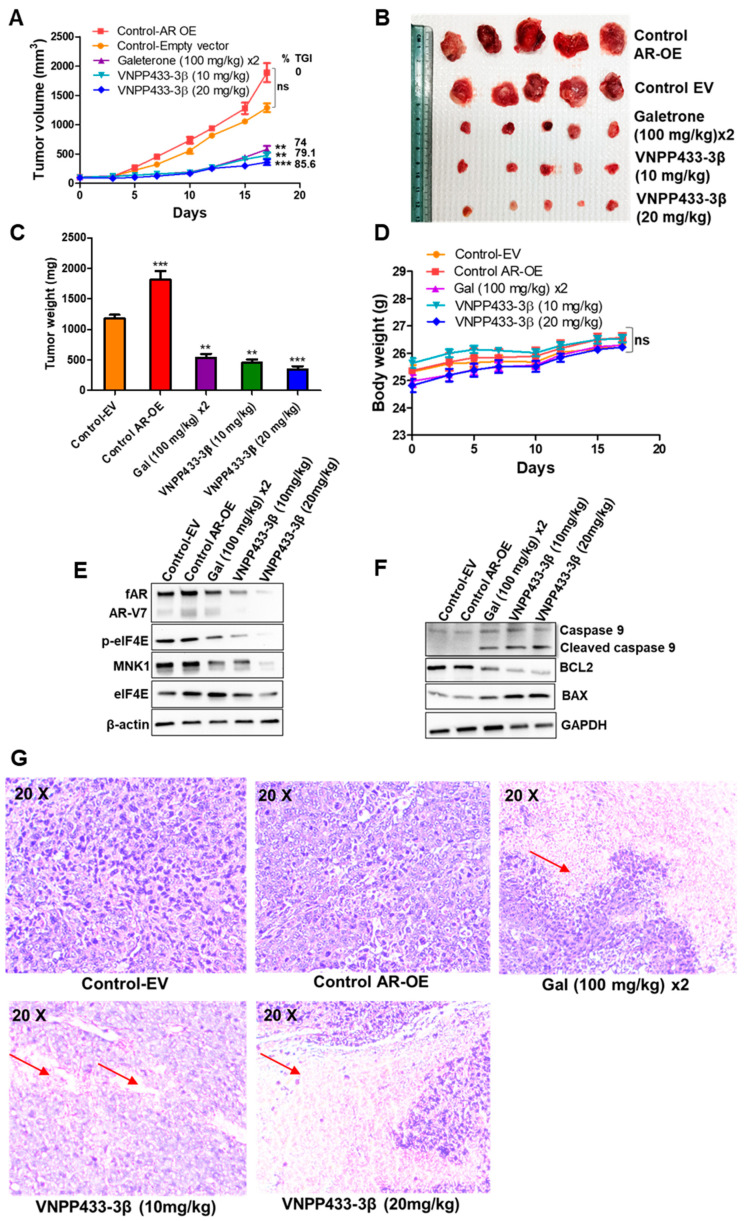
**Effect of VNPP433-3β in AR-over expressing 22Rv1 castration-resistant prostate cancer xenograft in vivo model.** (**A**) Tumor volume in the test animals: NRG mice bearing 22Rv1 tumor xenografts that stably overexpress AR were orally administered with vehicle (40% β-cyclodextrin in saline, PO), galeterone (100 mg/kg, twice a day) and VNPP433-3β (10 or 20 mg/kg)- 5 days a week for 17 days. Tumor size was measured thrice a week using digital calipers and volume was calculated using the formula length × width 2 × 0.5 (mm^3^) and plotted against time (days of treatment). Tumor growth inhibition (TGI) is calculated based on initial tumor volume (100%) and tumor volume in AR-OE control at the end of the experiment (0%). The results are represented as means ± SEM as error bar. ns *p* = 0.4056, ** *p* = 0.0030, *** *p* = 0.0001 (compared to Control AR-OE). (**B**) Excised tumors from all animals in each group were photographed. (**C**) Mean weight of excised tumor in each group with SE bars at the end of the study. (**D**) Mean body weight of animals during the study period in each group. (**E**) Western blot analyses of key proteins involved in mechanism of action of VNPP433-3β in the excised tumors. (**F**) Level of key apoptotic proteins in tumor samples affected by VNPP433-3β treatment. (**G**) Histological examination of excised tumors following Hematoxylin-Eosin (H and E) staining. Nuclei stains blue (large in number in proliferating control tumor) and cytoplasm/extracellular matrix stains pink (red arrows) indicative of tumor regression upon drug treatment.

Since the biochemical, cytological, and molecular data demonstrated AR as the prime target of VNPP433-3β, we sought to evaluate the effect of VNPP433-3β in AR-overexpressing tumor xenografts in vivo. The tumor xenografts from AR-overexpressing 22Rv1 were larger in volume and heavier in final weight than the empty vector-transfected 22Rv1 xenografts. However, when treated with galeterone (100 mg/kg, twice a day) or VNPP433-3β (10 or 20 mg/kg, once a day), five days a week for 17 days, the tumor growth was significantly impaired (Figure 6A–C). Interestingly, the effect of VNPP433-3β at 20 mg/kg body weight was found to be highly significant as indicated by the smallest tumor size among the treated groups with a calculated tumor growth inhibition (TGI) of 86% whereas galeterone (100 mg/kg) and VNPP433-3β at 10 mg/kg were comparable with a TGI of 74 and 79%, respectively. We did not observe any anomalous pattern in the body weight of the treated animals compared to the control over the study period, indicating that the test compounds do not have a significant adverse effect on the general health of the animals (Figure 6D). Interestingly, the western blot analyses of key target proteins in the excised tumors reveal that the level of AR was significantly reduced upon VNPP433-3β treatment in a concentration-dependent manner with a concomitant reduction in tumor volume and weight (Figure 6E). The apoptotic markers were also studied, reflecting an increase in BAX and a decrease in the antiapoptotic protein Bcl2. Cleaved Caspase 9 in treated tumor xenografts further confirms activation of apoptosis in tumor (Figure 6F). Further, the histologic examination revealed a reduction in tumor cells in tumors of treated animals (Figure 6G). This further affirms our cytological and biochemical data wherein VNPP433-3β was shown to directly engage AR in live cells of prostate cancer and mediates proteasome-dependent degradation of full-length AR and the clinically significant splice variant AR-V7. As demonstrated in vitro, the levels of Mnk1 and its target p-eIF4E were significantly reduced in tumor tissues of the galeterone/VNPP433-3β-treated animals.

## 4. Discussion

The goal of this study is to investigate the mechanism of action of VNPP433-3β in combating PCa. Our in vivo studies suggest that VNPP433-3β is more potent than galeterone in combating PCa [30]. In this study, we demonstrate that VNPP433-3β physically interacts with AR LBD in vitro and directly engages fAR within the live prostate cancer cells. The half-life of key oncoproteins is a crucial factor to endure the high metabolic demand in cancer cells. Typically, non-ligand bound AR is degraded by the proteasome, but androgen-binding stabilizes it and prolongs the half-life [41]. Notably, the half-life of fAR and AR-V7 was significantly decreased upon VNPP433-3β treatment. Previous studies using MG132 have shown a significant reduction in AR half-life when LNCaP cells were challenged by Gal [43,48]. Discovery and development of novel AR degraders could go a long way in offsetting drug resistance as demonstrated using PROTAC that degraded fAR and inhibited tumor cell growth even in models overexpressing AR-V7 [49]. However, the persistence of AR-V7 cannot be ignored as many studies implicate AR-V7 for enzalutamide/drug resistance in PCa, and resistance correlated with elevated levels of AR-V7 [50]. The present study establishes a major milestone in this direction as VNPP433-3β is demonstrated to trigger enhanced proteasomal degradation of fAR as well as AR-V7 and the effect was superior to that of Gal. Treating the cells with proteasome inhibitor MG132 averted VNPP433-3β-induced degradation of AR but lead to accumulation of ubiquitinated fAR and AR-V7. This confirms the involvement of VNPP433-3β in promoting ubiquitin-mediated proteasomal degradation of fAR and AR-V7 by enhancing its interaction with E3 ligases CHIP and MDM2 as demonstrated in the subsequent co-IP experiments. AR is known to be regulated by MDM2-mediated ubiquitination and subsequent proteasomal degradation [51]. Coimmunoprecipitation studies demonstrate that the binding of VNPP433-3β to AR enhances its interaction with E3 ligases CHIP and Mdm2. This observation is interesting as it further supports the model of enhanced proteasomal degradation of AR, mediated by VNPP433-3β. Therefore, different lines of experimental evidence strongly support the accelerated degradation of AR via ubiquitin-dependent proteasomal pathway. We observed that VNPP433-3β-bound AR exhibited more affinity for the E3 ligases CHIP and MDM2 and significantly impaired AR-HSP90 interaction. Reduced affinity for HSP90 further rendered AR susceptible to degradation. These findings are in line with previous studies in which HSP90 is found to be critical for the active conformation of androgen-bound AR for its nuclear transfer and transcriptional activity [52]. Our in vivo studies further affirms that AR is the prime target of VNPP433-3β in AR-overexpressing CRPC tumor xenografts as indicated by the remarkable reduction in levels of AR in excised tumors and concomitant tumor inhibition.

**Figure 7:**
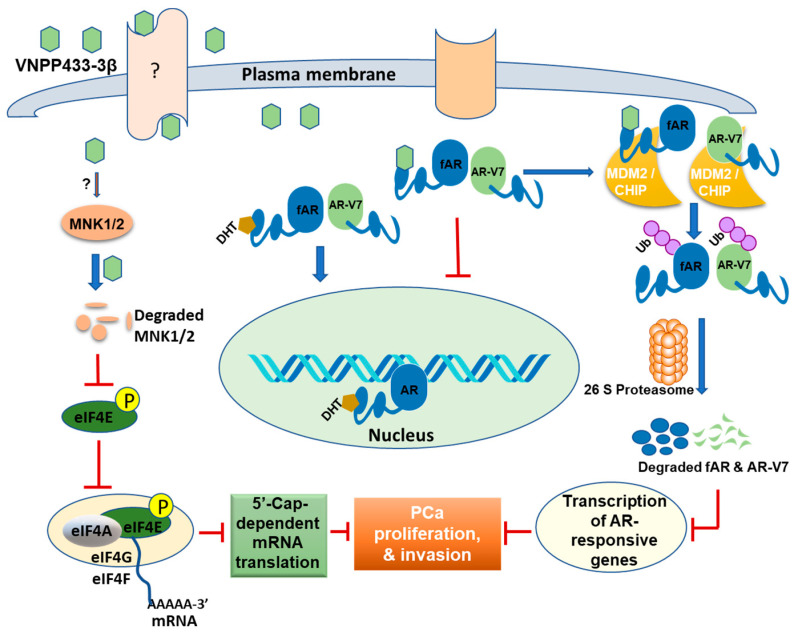
Graphical representation of the mechanism of action of VNPP433-3β in PCa inhibition. VNPP433-3β binds the AR, prevents its translocation into the nucleus, and redirects it to ubiquitination by MDM2/CHIP and subsequent degradation by 26 S proteasome thereby affecting transcription of AR-responsive tumor genes and affecting PCa inhibition. Additionally, VNPP433-3β impedes phosphorylation of eIF4E by depleting MNK1/2 that in turn limits 5′-cap-dependent translation and blocks PCa progression.

More notably, VNPP433-3β also triggers a significant reduction in the Mnk1/2 protein level. However, we are further investigating the mechanism of VNPP433-3β-induced Mnk1/2 depletion. Our findings are consistent with previous studies that showed phosphorylation of eIF4E by MNK1/2 is critical to promoting EMT and metastasis via translational control of SNAIL and MMP3 [53]. VNPP433-3β impedes reckless translation in PCa cells, a prerequisite to slowing down the active tumor proliferation by abating the binding of eIF4E and eIF4G to 5′ caps of mRNA. This presents a direct consequence for the translation of AR mRNA as well as other key proteins as studies have shown that level of AR protein is also regulated at the translational level by modulating the mRNA-5′cap-dependent translation [47]. Treating cells with VNPP433-3β also reduced the level of p-4EBP1 significantly and this correlates with the decreased eIF4E activity as unphosphorylated 4EBP1 sequesters eIF4E by direct binding [46]. However, phosphorylation of 4EBP1 by mTOR decreases its affinity for eIF4E and renders it available for translation initiation [25]. The molecular mechanism of action of VNPP433-3β that leads to PCa inhibition is schematically depicted in Figure 7.

Recent RNA-seq studies in PCa suggest that AR-V7 acts as a transcriptional repressor of tumor-suppressor genes [54]. Transcriptome analyses by RNA-seq present a global view of changes in gene expression profile in PCa. Transcriptome analyses of VNPP433-3β-treated PCa cells suggest that VNPP433-3β transcriptionally modulates more than 20 canonical pathways that are significantly associated with PCa and transcriptionally complement its biochemical effects on PCa inhibition. VNPP433-3β-mediated decreased transcription of AR further contributes to the decline in total AR levels in the cell. VNPP433-3β impedes androgen binding to the AR and thus prevents its activation into a functional transcription factor thereby affecting its own transcription as well as other genes regulated by AR.

Collectively, our data suggest that VNPP433-3β directly targets AR within the cells and inhibits mCRPC. VNPP433-3β also precludes eIF4E phosphorylation by depleting Mnk1/2 besides dodging binding of eIF4E and eIF4G to mRNA 5′ cap thereby impeding the reckless translational activity of the PCa cells. Finally, VNPP433-3β obstructs cell proliferation, colony formation, invasion, and EMT in PCa cells and enforces apoptosis. Transcriptome analyses by RNA-seq further substantiate the biochemical findings and reveal several pathways relevant to PCa that are modulated by VNPP433-3β. Notably, VNPP433-3β acts at multiple levels and modulates more than 20 cellular pathways to halt PCa progression and redirect the cells to undergo apoptosis. Based on the results of the present study, we propose that VNPP433-3β exerts the antitumor effect by imposing (1) transcriptional regulation of AR and AR-responsive oncogenes via degrading AR, (2) translational regulation by depleting Mnk1/2 thereby impeding phosphorylation of eIF4E and subsequent mRNA-5′cap-dependent translation initiation, (3) reducing AR half-life through enhanced proteasomal degradation and (4) modulating transcription of several genes relevant to PCa. As VNPP433-3β exerts its therapeutic effect at multiple levels, it could potentially be developed for clinical trials in mCRPC patients.

## 5. Conclusions

VNPP433-3β modulates AR-responsive oncogenic transcription by directly targeting AR and augmenting its degradation. VNPP433-3β-bound AR loses affinity for HSP90 and preferentially interacts with Mdm2 and CHIP resulting in its ubiquitination and subsequent proteasomal degradation. Further, it perturbs eIF4E phosphorylation by depleting Mnk1/2 besides disrupting the binding of eIF4E and eIF4G to mRNA 5′ cap, thereby upsetting reckless translational activity of the PCa cells. Finally, it modulates the transcription of several genes and activates more than 20 cellular pathways, that synergistically contribute to PCa inhibition. As VNPP433-3β effectively disrupts multiple cellular pathways that drive PCa development, progression, and metastasis, it could possibly thwart therapy resistance, making it an excellent candidate for clinical development.

## Data Availability

The datasets used and analyzed during the current study are available from the corresponding author on reasonable request.

## Supplementary Materials

The following supporting information can be downloaded at: https://www.mdpi.com/article/10.3390/cells11172699/s1, Table S1: Selected genes relevant to PCa that are upregulated (highlighted in orange) or downregulated (highlighted in blue) by VNPP433-3β as demonstrated by RNA-seq.

## References

[1] Siegel R.L., Miller K.D., Jemal A. (2020). Cancer statistics, 2020. CA Cancer J. Clin..

[2] Torre L.A., Bray F., Siegel R.L., Ferlay J., Lortet-Tieulent J., Jemal A. (2015). Global cancer statistics, 2012. CA Cancer J. Clin..

[3] Labriola M.K., Atiq S., Hirshman N., Bitting R.L. (2021). Management of men with metastatic castration-resistant prostate cancer following potent androgen receptor inhibition: A review of novel investigational therapies. Prostate Cancer Prostatic Dis..

[4] D’Abronzo L.S., Ghosh P.M. (2018). eIF4E Phosphorylation in Prostate Cancer. Neoplasia.

[5] Szostak M.J., Kyprianou N. (2000). Radiation-induced apoptosis: Predictive and therapeutic significance in radiotherapy of prostate cancer (review). Oncol. Rep..

[6] Schiewer M.J., Augello M.A., Knudsen K.E. (2012). The AR dependent cell cycle: Mechanisms and cancer relevance. Mol. Cell Endocrinol..

[7] Pilling A.B., Hwang C. (2019). Targeting prosurvival BCL2 signaling through Akt blockade sensitizes castration-resistant prostate cancer cells to enzalutamide. Prostate.

[8] Attard G., Parker C., Eeles R.A., Schröder F., Tomlins S.A., Tannock I., Drake C.G., de Bono J.S. (2016). Prostate cancer. Lancet.

[9] Watson P.A., Arora V.K., Sawyers C.L. (2015). Emerging mechanisms of resistance to androgen receptor inhibitors in prostate cancer. Nat. Rev. Cancer.

[10] Rossi V., Di Zazzo E., Galasso G., De Rosa C., Abbondanza C., Sinisi A.A., Altucci L., Migliaccio A., Castoria G. (2019). Estrogens Modulate Somatostatin Receptors Expression and Synergize With the Somatostatin Analog Pasireotide in Prostate Cells. Front. Pharmacol..

[11] Di Zazzo E., Galasso G., Giovannelli P., Di Donato M., Bilancio A., Perillo B., Sinisi A.A., Migliaccio A., Castoria G. (2019). Estrogen Receptors in Epithelial-Mesenchymal Transition of Prostate Cancer. Cancers.

[12] de Bono J.S., Logothetis C.J., Molina A., Fizazi K., North S., Chu L., Chi K.N., Jones R.J., Goodman O.B., Saad F. (2011). Abiraterone and increased survival in metastatic prostate cancer. N. Engl. J. Med..

[13] Ryan C.J., Smith M.R., De Bono J.S., Molina A., Logothetis C.J., De Souza P., Fizazi K., Mainwaring P., Piulats J.M., Ng S. (2013). Abiraterone in metastatic prostate cancer without previous chemotherapy. N. Engl. J. Med..

[14] Beer T.M., Armstrong A.J., Rathkopf D.E., Loriot Y., Sternberg C.N., Higano C.S., Iversen P., Bhattacharya S., Carles J., Chowdhury S. (2014). Enzalutamide in metastatic prostate cancer before chemotherapy. N. Engl. J. Med..

[15] Scher H.I., Fizazi K., Saad F., Taplin M.-E., Sternberg C.N., Miller K., De Wit R., Mulders P., Chi K.N., Shore N.D. (2012). Increased survival with enzalutamide in prostate cancer after chemotherapy. N. Engl. J. Med..

[16] Mohler J.L., Gregory C.W., Ford O.H., Kim D., Weaver C.M., Petrusz P., Wilson E.M., French F.S. (2004). The androgen axis in recurrent prostate cancer. Clin. Cancer Res..

[17] Carreira S., Romanel A., Goodall J., Grist E., Ferraldeschi R., Miranda S., Prandi D., Lorente D., Frenel J.-S., Pezaro C. (2014). Tumor clone dynamics in lethal prostate cancer. Sci. Transl. Med..

[18] Visakorpi T., Hyytinen E., Koivisto P., Tanner M., Keinänen R., Palmberg C., Palotie A., Tammela T., Isola J., Kallioniemi O.P. (1995). In vivo amplification of the androgen receptor gene and progression of human prostate cancer. Nat. Genet..

[19] Huang Z.Q., Li J., Wong J. (2002). AR possesses an intrinsic hormone-independent transcriptional activity. Mol. Endocrinol..

[20] Zegarra-Moro O.L., Schmidt L.J., Huang H., Tindall N.J. (2002). Disruption of androgen receptor function inhibits proliferation of androgen-refractory prostate cancer cells. Cancer Res..

[21] Balk S.P. (2002). Androgen receptor as a target in androgen-independent prostate cancer. Urology.

[22] Balk S.P., Knudsen K.E. (2008). AR, the cell cycle, and prostate cancer. Nucl. Recept. Signal..

[23] Njar V.C.O. (2017). Androgen receptor antagonism and impact on inhibitors of androgen synthesis in prostate cancer therapy. Transl. Cancer Res..

[24] Sarkar S., Brautigan D.L., Parsons S.J., Larner J.M. (2014). Androgen receptor degradation by the E3 ligase CHIP modulates mitotic arrest in prostate cancer cells. Oncogene.

[25] D’Abronzo L.S., Bose S., Crapuchettes M.E., Beggs R.E., Vinall R.L., Tepper C.G., Siddiqui S., Mudryj M., Melgoza F.U., Durbin-Johnson B.P. (2017). The androgen receptor is a negative regulator of eIF4E phosphorylation at S209: Implications for the use of mTOR inhibitors in advanced prostate cancer. Oncogene.

[26] Pyronnet S., Imataka H., Gingras A.-C., Fukunaga R., Hunter T., Sonenberg N. (1999). Human eukaryotic translation initiation factor 4G (eIF4G) recruits mnk1 to phosphorylate eIF4E. EMBO J..

[27] Njar V.C., Brodie A.M. (2015). Discovery and development of Galeterone (TOK-001 or VN/124-1) for the treatment of all stages of prostate cancer. J. Med. Chem..

[28] Montgomery B., Eisenberger M.A., Rettig M.B., Chu F., Pili R., Stephenson J.J., Vogelzang N.J., Koletsky A.J., Nordquist L.T., Edenfield W.J. (2016). Androgen Receptor Modulation Optimized for Response (ARMOR) Phase I and II Studies: Galeterone for the Treatment of Castration-Resistant Prostate Cancer. Clin. Cancer Res..

[29] Purushottamachar P., Thomas E., Thankan R.S., Rudchenko V., Hualng G., Njar V.C. (2022). Large-scale synthesis of galeterone and lead next generation galeterone analog VNPP433-3beta. Steroids.

[30] Kwegyir-Afful A.K., Ramalingam S., Ramamurthy V.P., Purushottamachar P., Murigi F.N., Vasaitis T.S., Huang W., Kane M.A., Zhang Y., Ambulos N. (2019). Galeterone and The Next Generation Galeterone Analogs, VNPP414 and VNPP433-3beta Exert Potent Therapeutic Effects in Castration-/Drug-Resistant Prostate Cancer Preclinical Models In Vitro and In Vivo. Cancers.

[31] Thomas E., Thankan R.S., Purushottamachar P., Huang W., Kane M.A., Zhang Y., Ambulos N., Weber D.J., Njar V.C.O. (2022). Transcriptome profiling reveals that VNPP433-3β, the lead next-generation galeterone analog, inhibits prostate cancer stem cells by downregulating EMT and stem cell markers. Mol. Carcinog..

[32] Purushottamachar P., Kwegyir-Afful A.K., Martin M.S., Ramamurthy V.P., Ramalingam S., Njar V.C.O. (2016). Identification of Novel Steroidal Androgen Receptor Degrading Agents Inspired by Galeterone 3beta-Imidazole Carbamate. ACS Med. Chem. Lett..

[33] Matias P.M., Donner P., Coelho R., Thomaz M., Peixoto C., Macedo S., Otto N., Joschko S., Scholz P., Wegg A. (2000). Structural evidence for ligand specificity in the binding domain of the human androgen receptor. Implications for pathogenic gene mutations. J. Biol. Chem..

[34] Trott O., Olson A.J. (2010). AutoDock Vina: Improving the speed and accuracy of docking with a new scoring function, efficient optimization, and multithreading. J. Comput. Chem..

[35] Thomas E., Gopalakrishnan V., Hegde M., Kumar S., Karki S.S., Raghavan S.C., Choudhary B. (2016). A Novel Resveratrol Based Tubulin Inhibitor Induces Mitotic Arrest and Activates Apoptosis in Cancer Cells. Sci. Rep..

[36] Jafari R., Almqvist H., Axelsson H., Ignatushchenko M., Lundbäck T., Nordlund P., Molina D.M. (2014). The cellular thermal shift assay for evaluating drug target interactions in cells. Nat. Protoc..

[37] Purushottamachar P., Thomas E., Thankan R.S., Njar V.C. (2022). Novel deuterated Mnk1/2 protein degrader VNLG-152R analogs: Synthesis, In vitro Anti-TNBC activities and pharmacokinetics in mice. Eur. J. Med. Chem..

[38] Thomas E., Gopalakrishnan V., Somasagara R.R., Choudhary B., Raghavan S.C. (2016). Extract of Vernonia condensata, Inhibits Tumor Progression and Improves Survival of Tumor-allograft Bearing Mouse. Sci. Rep..

[39] Hegde M., Karki S.S., Thomas E., Kumar S., Panjamurthy K., Ranganatha S.R., Rangappa K.S., Choudhary B., Raghavan S.C. (2012). Novel levamisole derivative induces extrinsic pathway of apoptosis in cancer cells and inhibits tumor progression in mice. PLoS ONE.

[40] Liang J., Wang L., Poluben L., Nouri M., Arai S., Xie L., Voznesensky O.S., Cato L., Yuan X., Russo J.W. (2021). Androgen receptor splice variant 7 functions independently of the full length receptor in prostate cancer cells. Cancer Lett..

[41] Sheflin L., Keegan B., Zhang W., Spaulding S.W. (2000). Inhibiting proteasomes in human HepG2 and LNCaP cells increases endogenous androgen receptor levels. Biochem. Biophys. Res. Commun..

[42] Bruno R.D., Gover T.D., Burger A.M., Brodie A.M., Njar V.C. (2008). 17alpha-Hydroxylase/17,20 lyase inhibitor VN/124-1 inhibits growth of androgen-independent prostate cancer cells via induction of the endoplasmic reticulum stress response. Mol. Cancer Ther..

[43] Yu Z., Cai C., Gao S., Simon N.I., Shen H.C., Balk S.P. (2014). Galeterone prevents androgen receptor binding to chromatin and enhances degradation of mutant androgen receptor. Clin. Cancer Res..

[44] Cnop M., Toivonen S., Igoillo-Esteve M., Salpea P. (2017). Endoplasmic reticulum stress and eIF2alpha phosphorylation: The Achilles heel of pancreatic beta cells. Mol. Metab..

[45] Shafi A.A., Cox M.B., Weigel N.L. (2013). Androgen receptor splice variants are resistant to inhibitors of Hsp90 and FKBP52, which alter androgen receptor activity and expression. Steroids.

[46] Karaki S., Andrieu C., Ziouziou H., Rocchi P. (2015). The Eukaryotic Translation Initiation Factor 4E (eIF4E) as a Therapeutic Target for Cancer. Adv. Protein Chem. Struct. Biol..

[47] Cinar B., de Benedetti A., Freeman M.R. (2005). Post-transcriptional regulation of the androgen receptor by Mammalian target of rapamycin. Cancer Res..

[48] Kwegyir-Afful A.K., Ramalingam S., Purushottamachar P., Ramamurthy V.P., Njar V.C.O. (2015). Galeterone and VNPT55 induce proteasomal degradation of AR/AR-V7, induce significant apoptosis via cytochrome c release and suppress growth of castration resistant prostate cancer xenografts in vivo. Oncotarget.

[49] Kregel S., Wang C., Han X., Xiao L., Fernandez-Salas E., Bawa P., McCollum B.L., Wilder-Romans K., Apel I.J., Cao X. (2020). Androgen receptor degraders overcome common resistance mechanisms developed during prostate cancer treatment. Neoplasia.

[50] Zhu Y., Dalrymple S.L., Coleman I., Zheng S.L., Xu J., Hooper J.E., Antonarakis E.S., De Marzo A.M., Meeker A.K., Nelson P.S. (2020). Role of androgen receptor splice variant-7 (AR-V7) in prostate cancer resistance to 2nd-generation androgen receptor signaling inhibitors. Oncogene.

[51] Gaughan L., Logan I.R., Neal D.E., Robson C.N. (2005). Regulation of androgen receptor and histone deacetylase 1 by Mdm2-mediated ubiquitylation. Nucleic. Acids Res..

[52] Georget V., Térouanne B., Nicolas J.-C., Sultan C. (2002). Mechanism of antiandrogen action: Key role of hsp90 in conformational change and transcriptional activity of the androgen receptor. Biochemistry.

[53] Robichaud N., del Rincon S.V., Huor B., Alain T., Petruccelli L.A., Hearnden J., Goncalves C., Grotegut S., Spruck C.H., Furic L. (2015). Phosphorylation of eIF4E promotes EMT and metastasis via translational control of SNAIL and MMP-3. Oncogene.

[54] Cato L., de Tribolet-Hardy J., Lee I., Rottenberg J.T., Coleman I., Melchers D., Houtman R., Xiao T., Li W., Uo T. (2019). ARv7 Represses Tumor-Suppressor Genes in Castration-Resistant Prostate Cancer. Cancer Cell.

